# Inhibition of STAT3 signaling and induction of SHP1 mediate antiangiogenic and antitumor activities of ergosterol peroxide in U266 multiple myeloma cells

**DOI:** 10.1186/1471-2407-12-28

**Published:** 2012-01-20

**Authors:** Yun-Hee Rhee, Soo-Jin Jeong, Hyo-Jeong Lee, Hyo-Jung Lee, Wonil Koh, Ji Hoon Jung, Sun-Hee Kim, Kim Sung-Hoon

**Affiliations:** 1 Clinical Trial Institute, Dankook University, Chenan 330-714, South Korea; 2 College of Oriental Medicine, Kyung Hee University, Seoul 130-701, South Korea

**Keywords:** ergosterol peroxide, JAK2, STAT3, angiogenesis, multiple myeloma

## Abstract

**Background:**

Ergosterol peroxide (EP) derived from edible mushroom has been shown to exert anti-tumor activity in several cancer cells. In the present study, anti-angiogenic activity of EP was investigated with the underlying molecular mechanisms in human multiple myeloma U266 cells.

**Results:**

Despite weak cytotoxicity against U266 cells, EP suppressed phosphorylation, DNA binding activity and nuclear translocalization of signal transducer and activator of transcription 3 (STAT3) in U266 cells at nontoxic concentrations. Also, EP inhibited phosphorylation of the upstream kinases Janus kinase 2 (JAK2) and Src in a time-dependent manner. Furthermore, EP increased the expression of protein tyrosine phosphatase SHP-1 at protein and mRNA levels, and conversely silencing of the SHP-1 gene clearly blocked EP-mediated STAT3 inactivation. In addition, EP significantly decreased vascular endothelial growth factor (VEGF), one of STAT3 target genes at cellular and protein levels as well as disrupted *in vitro *tube formation assay. Moreover, EP significantly suppressed the growth of U266 cells inoculated in female BALB/c athymic nude mice and immunohistochemistry revealed that EP effectively reduced the expression of STAT3 and CD34 in tumor sections compared to untreated control.

**Conclusion:**

These findings suggest that EP can exert antitumor activity in multiple myeloma U266 cells partly with antiangiogenic activity targeting JAK2/STAT3 signaling pathway as a potent cancer preventive agent for treatment of multiple myeloma cells.

## Background

Ergosterol Peroxide (EP), 5α, 8α-epidioxy-22E-ergosta-6, 22-dien-3β-ol, is found in plants [1], lichens [2] and mushrooms such as *Ganoderma lucidum *[3], *Sporothrix schenckii *[4] and *Cordyceps sinensis *[5]. Despite various biological effects of EP such as immunosuppressive [6-8], anti-viral [9], anti-inflammatory [10] and anti-tumor [5,10] activities, the underlying molecular mechanisms for anti-cancer activity of EP still remain unclear.

STAT proteins originally discovered as latent cytoplasmic transcription factors [11] are involved in a variety of cellular processes such as cell proliferation, differentiation and apoptosis [12,13]. Of the STAT proteins, STAT3 is often constitutively activated in many human cancer cells including multiple myeloma, leukemia, lymphoma, and solid tumors [14,15]. Upon activation, STAT3 undergoes phosphorylation-induced homodimerization, leading to nuclear translocation, DNA binding and subsequent gene transcription. STAT3 phosphorylation is mediated through the activation of non-receptor protein tyrosine kinases Janus activated kinases (JAKs) and c-Src [16].

STAT3 participates in oncogenesis through up-regulation of genes encoding apoptosis inhibitors such as bcl-x_L_, bcl-2, and survivin [15]. Also, recent studies reported the evidences that STAT3 is involved in the regulation of angiogenesis through modulation of VEGF, a key regulator of angiogenesis [17-19]. In this regards, we investigated anti-angiogenic effect of EP in association with JAK2/STAT3 pathway and STAT3 related VEGF expression in U266 cells *in vitro *and mouse xenograft model.

## Methods

### Isolation of ergosterol peroxide (EP)

EP was isolated according to the Krzyczkowski's method as previously described [20].

### Cell culture

U266 (multiple myeloma), SCC4 (head and neck squamous cell carcinoma), DU145 (prostate cancer), and MDA-MB-231 (breast cancer) cells were obtained from American Type Culture Collection (ATCC) (Rockville, MD) and maintained in RPMI 1640 supplemented with 10% FBS and antibiotics. Human umbilical vein endothelial cells (HUVECs) were isolated from fresh human umbilical cord vein by collagenase treatment as described previously [21]. The cells were maintained in in EBM-2 containing 2% FBS, 0.04% hydrocortisone, 0.4% hFGF-B, 0.1% VEGF, 0.1% IGF-1, 0.1% ascorbic acid, 0.1% hEGF, 1% GA-1000, and 1% Heparin (Walkersville, MD)

### Cytotoxicity assay

The cytotoxic effect of EP was evaluated by 3-(4, 5-dimethylthiazol-2-yl)-2, 5-diphenyltetrazolium bromide (MTT) assay. U266 cells were seeded onto 96-well microplate at a density of 1 × 10^4 ^cells/well and treated with various concentrations of EP (0, 6.25, 12.5, 25 or 50 μM) for 24 h. The culture medium was removed after centrifuging the plate and MTT solution (1 mg/ml) was added to each well until formazan was constituted. MTT lysis solution (20% SDS and 50% dimethylfermamide) was added to dissolve formazan and optical density (OD) was measured using microplate reader (Tecan Austria GmbH, Grödig, Austria) at 570 nm. Cell viability was calculated as a percentage of viable cells in EP-treated group versus untreated control by following equation.

$$\text{Cell viability}\left(\%\right)=\left[\text{OD}\;\left(\text{EP}\right)-\text{OD}\;\left(\text{Blank}\right)\right]/\left[\text{OD}\;\left(\text{Control}\right)-\text{OD}\left(\text{Blank}\right)\right]\times \text{1}00$$

### Western blotting

Cells lysates were prepared using lysis buffer [50 mM Tris (pH 7.4), 300 mM NaCl, 5 mM EDTA (pH 8.0), 0.5% Triton X-100, 1 mM aprotinin, 1 mM leupeptin, 1 mM pepstatin, 10 mM iodoacetamide, 2 mM phenylmethylsulfonyl fluoride (PMSF), and 1 mM NaVO_4_] and centrifuged at 14, 000 × g for 10 min at 4°C. Protein samples were collected, separated onto 10-12% SDS-PAGE gels and electrotransferred to a nitrocellulose membrane. The membranes were blocked in 5% nonfat skim milk, probed with primary antibodies for STAT3, phospho-STAT3^Y705^, JAK2, phospho-JAK2^Y1007/1008^, Src, phospho-Src^Y416 ^(Cell Signaling, Danvers, MA), SHP-1, VEGF (Santa Cruz Biotechnology, Santa Cruz, CA) and β-actin (Sigma, St. Louis, MO) at 4°C for overnight, and then exposed to HRP-conjugated secondary antibodies at room temperature for 2 h. Protein expression was detected by using enhanced chemiluminescence (ECL) kit (Amersham Pharmacia, Piscataway, NJ).

### Electrophoretic mobility shift assay (EMSA) for STAT3-DNA binding

The STAT3-DNA binding was analyzed by electrophoretic mobility shift assay (EMSA) using a ^32^P-labeled high-affinity sis-inducible element (hSIE) probe (5'-CTTCATTTCCCGTAAATCCCTAAAGCT-3' and 5'-AGCTTTAGGGATTTACGG GAAA TGA-3') as previously described [22]. Briefly, nuclear extracts were incubated with the hSIE probe and the protein-DNA complexes were separated onto 5% native polyacrylamide gels. The gels were dried, and the radioactive bands were quantitated with Storm 820 and Imagequant software (Amersham Pharmacia, Piscataway, NJ).

### RT-PCR analysis

Total RNA was extracted by using Trizol reagent (Invitrogen) according to the manufacturer's instructions. cDNA was synthesized from 1 μg of total RNA and subjected to PCR reaction by using Superscript One Step reverse transcription-PCR (RT-PCR) kit (Invitrogen). The PCR conditions were 30 cycles of 94°C for 15 s, 55°C for 30 s, and 72°C for 1 min. The primer sequences were as follows: *SHP-1 *(forward primer 5'-AAT GCG TCC CAT ACT GGC CCG A-3'; reverse primer 5'-CCC GCA GTT GGT CAC AGA GT-3'), and *GAPDH *(forward primer 5'-TCA CCA TCT TCC AGG AGC GA-3'; reverse primer 5'-CAC AAT GCC GAA GTG GTG GT-3').

### siRNA transfection

siRNA oligonucleotides for SHP-1 (SantaCruz biotechnology, SantaCruz, CA) were transfected by using LipofectAMINE transfection reagent (Invitrogen, Carlsbad, CA) according to manufacturer's protocols.

### Enzyme- linked immunosorbent assay (ELISA) for VEGF

U266 cells were seeded onto 6-well plates at a density of 1 × 10^6 ^cells/well and treated with 25 μM EP. The VEGF levels in the supernatant were measured by using a Quantikine VEGF ELISA kit (R&D systems, Minneapolis, MN) according to manufacturer's protocols.

### In vitro tube formation assay

Tube formation assay was performed on Matrigel (Becton Dickinson Labware, Bedford, MA) as described previously [23]. In brief, human umbilical vein endothelial cells (HUVECs) (3 × 10^4 ^cells/well) were seeded onto Matrigel-coated 24-well plates and treated with VEGF (20 ng/ml) in the absence or presence of EP (0, 10 or 25 μM) for 6 h. Cells were fixed with 2% paraformaldehyde and stained with 2% crystal violet. Tube formation was observed under an Axiovert S 100 light microscope (Carl Zeiss, Weimar, Germany) and counted in randomly selected areas using NIH Scion image program.

### Mouse xenograft model

Six-week-old female athymic nude mice were purchased from Jung Ang lab animal (Seoul, Republic of Korea) and maintained under conventional conditions. U266 cells (2 × 10^6 ^cells) were mixed with Matrigel (Becton Dickinson, 50%, in 100 μl) and injected subcutaneously on the right flank of the mice. After 5 days of inoculation, the mice (n = 5/group) were given intraperitonial (i.p) injection of EP at 100 mg/Kg in 2% tween-80, or bortezomib *i.p*. 0.25 mg/kg in PBS every 2 or 3 days. Control mice were administered the solvent vehicle. Tumor volume was measured every other day with caliper and calculated according to the formula; V = 0.25*a*^2^*b*, where *a *is the smallest superficial diameter and *b *is the largest superficial diameter.

### Immunohistochemistry

The animals were sacrificed 20 days after U266 inoculation, and tumors were immediately removed, fixed in 4% paraformaldehyde, paraffin-embedded, and sectioned at 4 μm. Immunohistochemical staining for phospho-STAT3(Cell Signaling Technology, Danvers, MA), CD 34 (microvessel marker, Abcam, Boston, MA) and TUNEL (Calbiochem, Darmstadt, Germany) was performed and detected by DAB substrate staining (brown).

### Statistical analysis

All values were expressed as means ± S.D. Data were analyzed by one-way analysis of variance (ANOVA) and by student's t-test (Sigma plot^®^, San Rafael, CA, USA).

## Results

### Ergosterol peroxide suppresses STAT3 activation in U266 cells

**Figure 1:**
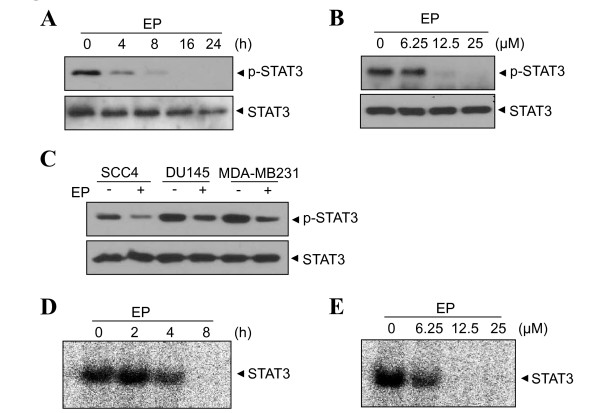
**Ergosterol peroxide (EP) suppresses STAT3 activation in U266 cells**. (A) Cells were treated with 25 μM EP for 0, 4, 8, 16 or 24 h. (B) Cells were treated with various concentrations of EP (0, 6.25, 12.5 or 25 μM) for 8 h. (C) SCC4, DU145 and MDA-MB-231 cells were treated with or without EP (25 μM) for 8 h. Whole cell extracts were prepared and subjected to Western blotting to determine level of phospho-STAT3 and STAT3. (D and E) Gel shift mobility assay was performed using nuclear extracts to examine the STAT3-DNA binding activity.

STAT3 is implicated in cell survival and growth in multiple myeloma cells [14]. In light of this event, we examined whether EP can modulate the constitutive activation of STAT3 in U266 cells. EP significantly inhibited the constitutive activation of STAT3 in time- and dose-dependent manners (Figure 1A and 1B, respectively). Tyrosine phosphorylation of STATs causes their dimerization and translocation to the nucleus, where they bind to DNA and regulate gene transcription [22]. Furthermore, we evaluated whether EP can affect on constitutive STAT3 activation in other cancer cell lines. SCC4 (head and neck squamous cell carcinoma), DU145 (prostate cancer), and MDA-MB-231 (breast cancer) cells expressing constitutive STAT3 were treated with or without EP. As shown in Figure 1C, EP inhibited STAT3 activation without affecting total STAT3 level. Consistent with the results of Western blotting in Figure 1A and 1B, gel shift assay showed that EP clearly inhibited the STAT3 DNA-binding activity in time- and dose-dependent manners (Figure 1D and 1E, respectively).

### Ergosterol peroxide suppresses JAK2 and Src in U266 cells

**Figure 2:**
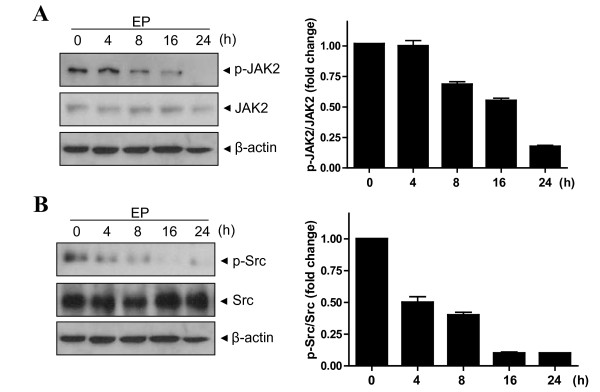
**Ergosterol peroxide (EP) inhibits phosphorylation of JAK2 and Src in U266 cells**. Cells were treated with 25 μM EP for 0, 4, 8, 16 or 24 h. Whole cell extracts were prepared and subjected to Western blotting to determine level of (A) phospho-JAK2 and JAK2, adn (B) phospho-Src and Src.

STAT3 is activated by soluble tyrosine kinases JAKs [24]. Effect of EP was measured on the activation of JAK2 by Western blotting. EP suppressed the phosphorylation of JAK2 in a time-dependent manner (Figure 2A), while it did not change the level of JAK1 (data not shown). The Src is another tyrosine kinase family to activate STAT3 signaling [16]. EP also decreased the phosphorylation of Src in a time-dependent manner (Figure 2B). These results indicate that EP inactivates the upstream kinases JAK2 and Src of STAT3 in U266 cells.

### Ergosterol peroxide enhances expression of SHP-1 in U266 cells

**Figure 3:**
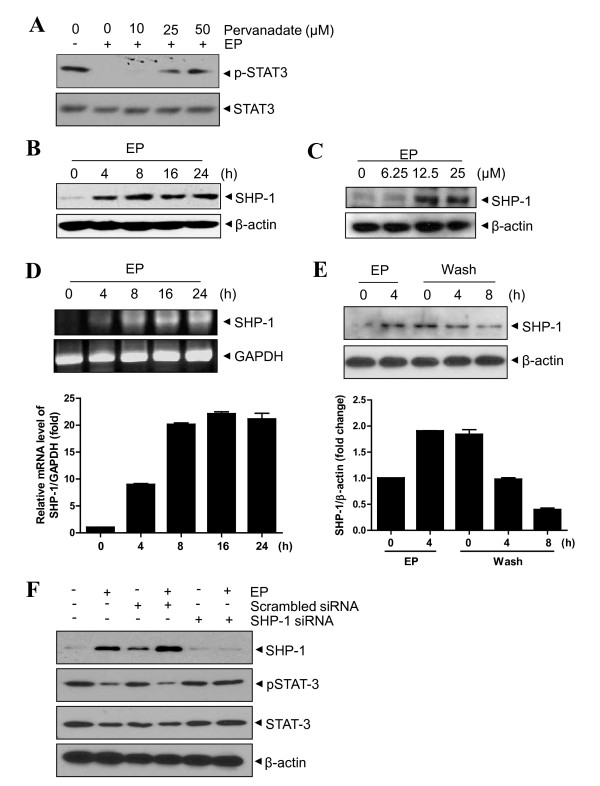
**Ergosterol peroxide (EP) enhances expression of SHP-1 in U266 cells**. (A) Cells were treated with 25 μM EP in the absence or presence of pervanadate (10, 25 or 50 μM) for 4 h. Whole cell extracts were prepared and subjected to Western blotting to determine level of phospho-STAT3 and STAT3. (B and C) Cells were treated with 25 μM EP for 0, 4, 8, 16 or 24 h (B) and various concentrations of EP (0, 6.25, 12.5 or 25 μM) for 4 h (C). Whole cell extracts were prepared and subjected to Western blotting to determine SHP-1 expressoin. (D) Cells were treated with 25 μM EP for 0, 4, 8, 16 or 24 h. RT-PCR was performed to analyze mRNA expression of SHP-1. (E) Cells were treated with 25 μM EP for 4 h (left), or treated with 25 μM EP for 1 h followed by washing PBS to remove EP and resuspension in fresh medium (right). Western blotting was performed for SHP-1. (F) Cells were transiently transfected with either SHP-1 or scrambled siRNA (50 nM) for 48 h and then treated with 25 μM EP for 4 h. Western blotting was performed for phospho-STAT3 and STAT3.

Non-transmembrane protein tyrosine phosphatases (PTPs) play important roles in the negative regulation of the JAK/STAT signaling [25]. Thus, we investigated whether PTPs are involved in EP-mediated STAT3 inactivation using a PTP inhibitor sodium pervanadate. As shown in Figure 3A, pervanadate reversed EP-induced STAT3 inactivation in a dose-dependent manner, indicating the important role of PTP(s) in EP induced STAT3 inactivation in U266 cells.

Since SHP-1 is a PTP expressed most abundantly in hematopoietic cells [26,27], the effect of EP on the expression of SHP-1 was examined. EP enhanced SHP-1 protein expression in time- and dose-dependent manners (Figure 3B and 3C). Consistently, EP increased the expression of SHP-1 at the mRNA level in a time-dependent manner (Figure 3D). Conversely, EP-mediated SHP-1 was reduced in a time course by the removal of EP containing media, indicating that SHP-1 expression by EP is reversible (Figure 3E). Furthermore, blocking SHP-1 using its specific siRNA clearly abolishes the ability of EP to inhibit STAT3 while scrambled siRNA had no effect on the expression (Figure 3F). Taken together, these results showed evidence that SHP-1 plays a critical role in the suppression of STAT3 phosphorylation by EP.

### Ergosterol peroxide (EP) exerts anti-angiogenic activity in vitro

**Figure 4:**
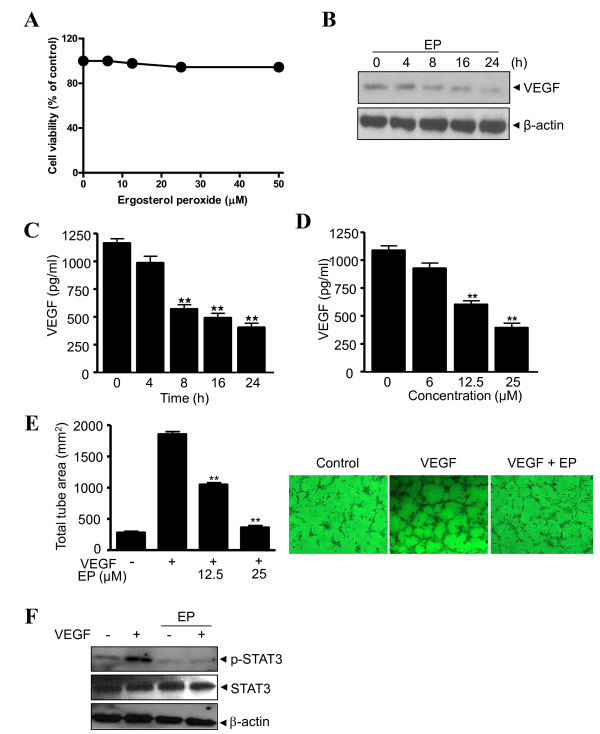
**Ergosterol peroxide (EP) exerts anti-angiogenic activity *in vitro***. (A) U266 cells were treated with various concentrations of EP (0, 6.25, 12.5, 25 or 50 μM) for 24 h. Cytotoxic effect of EP was evaluated by MTT assay. (B) U266 cells were treated with 25 μM EP for 0, 4, 8, 16 or 24 h. Cell lysates were prepared and subjected to Western blotting for VEGF. (C and D) U266 cells were seeded at density of 1 × 10^6 ^cells/well onto 6-well plates and treated with 25 μM EP for 0, 4, 8, 16 or 24 h (C) and various concentrations of EP (0, 6, 12.5 or 25 μM) for 24 h (D). Levels of VEGF in the supernatants were measured by using a Quantikine VEGF ELISA kit. (E) For *in vitro *tube formation assay, HUVECs (3 × 10^4 ^cells/well) were seeded onto Matrigel coated 24-well plates and treated with VEGF (20 ng/ml) in the absence or presence of EP (0, 12.5 or 25 μM) for 6 h. Cells were fixed with 2% paraformaldehyde, stained with 2% crystal violet, and the number of tube was randomly counted in selected areas (left). Representative photographs of tube formation in cells treated with VEGF (20 ng/ml) in the absence or presence of 25 μM EP (right). Each experiment was repeated three times and all data were expressed as means ± S.D. **, p < 0.01 *vs *untreated control. (F) HUVECs were treated with VEGF (20 ng/ml) in the absence or presence of 25 μM EP. Western blotting was performed for phopho-STAT3 and STAT3.

Cytotoxicity of EP against U266 cells were determined by MTT assay. U266 cells were treated with various concentrations of EP (0, 6.25, 12.5, 25 or 50 μM) for 24 h. As shown in Figure 4A, EP had no significant cytotoxic effect in U266 cells. STAT3 contributes to angiogenic regulation by inducing VEGF expression [28,29]. Thus, VEGF protein expression was analyzed at the cellular and protein levels in EP-treated cells. EP suppressed protein expression of VEGF in a time-dependent manner by Western blotting (Figure 4B). Consistently, secretion of VEGF was significantly inhibited by EP treatment in time- and dose-dependent manners by ELISA (Figure 4C and 4D, respectively).

To further confirm the anti-angiogenic activity of EP, *in vitro *tube formation assay was conducted using HUVECs treated either with or without VEGF in the absence or presence of EP. As shown in Figure 4E, no tube formation was observed in untreated cells, while clear tube formation was exhibited in VEGF-treated control. Notably, EP treatment inhibited VEGF-induced tube formation in a dose-dependent manner. Then, we tested whether EP can affect VEGF-mediated STAT3 activation in HUVECs. As shown in Figure 4F, VEGF treatment clearly enhanced phosphorylation of STAT3 in HUVECs, which was supported by Ebrahem's evidences [30]. In contrast, total STAT3 levels were not significantly changed by the treatment of VEGF and/or EP (Figure 4F).

### Ergosterol peroxide exerts anti-tumor activity in vivo

**Figure 5:**
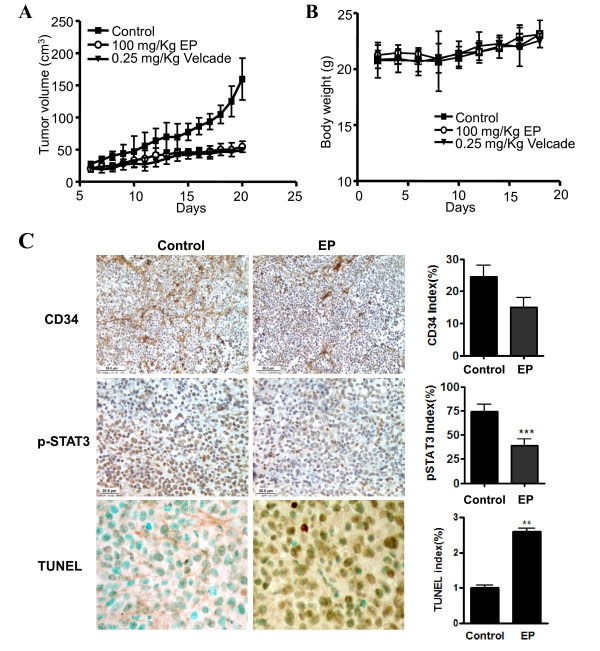
**Ergosterol peroxide (EP) exerts anti-tumor activity *in vivo***. Six-week-old female BALB/c athymic nude mice were subcutaneously injected in the flank area with 2 × 10^6 ^cells of U266 in 100 μl of matrigel mixed PBS. Five days after inoculation, mice (n = 5/group) were each given every 2-3 day intraperitonial (i.p.) injection of EP at 100 mg/kg in 2% tween-80 or bortezomib at 0.25 mg/kg in PBS. Control mice were administered the solvent vehicle. Tumor volumes were measured every other day with caliper. (A) Body weight was measured every other day after administration of EP or bortezomib. (B) Tumor volume was calculated according to the formula V = 0.25*a*^2^*b*, where *a *is the smallest superficial diameter and *b *is the largest superficial diameter. (C) Mice were killed on day 20 after cell inoculation, and tumors were immediately removed, fixed, embedded and sectioned at 4 μm for immunostaining of biomarkers. Representative photomicrographs of apoptosis detected by immunostaining of CD34, phospho-STAT3 and TUNEL at ×400, 200 and 100 of magnification, respectively. The sections were detected as DAB substrate staining (brown) and counterstained with Mayer's hematoxylin solution (right). Graphs show the CD34 index (angiogenesis), TUNEL index (apoptosis) and, pSTAT3 index in tumor sections. **p < 0.01 and ***p < 0.001 compared with vehicle-treated control mice (left).

To confirm the anti-tumor efficacy of EP, U266 cells were subcutaneously inoculated into BALB/c athymic nude mice in the flank area, and starting after 5 days injection, the mice were administered EP or bortezomib (a positive control) every 2-3 day with intraperitonial (*i.p*.) injection. Tumor growth was monitored every other day for 20 days. Tumor volumes in EP- or bortezomib-treated groups were decreased compared with untreated control (Figure 5A) without significant body weight loss in EP-treated group (Figure 5B).

Consistently, immunohistochemistry showed that EP treatment reduced the expression of phospho-STAT3 and angiogenesis marker CD34 compared to untreated control (Figure 5C), implying the antitumor effect of EP is associated with regulation of angiogenesis and STAT3 *in vitro *and *in vivo*. In addition, TUNEL positive cells for apoptosis were effectively increased in EP-treated tumor sections. However, considering that EP treatment for 48 h or 72 h exerted significant cytotoxicity against U266 cells, we can postulate that EP can exert antitumor effects via antiangiogenic activity at low concentration and apoptotic activity at higher concentration.

## Discussion

Multiple myeloma, also called plasma cells myeloma, is characterized by accumulation of secretory plasma cells. For the treatment of multiple myeloma, various chemotherapeutic agents such as vincristine, carmustine, mephalan, cyclophosphamide, doxorubicin, prednisone and dexamethasone have been used in combination or either drug alone [31]. However, many multiple myeloma patients frequently had no response to these agents and their prolonged exposure induced toxicity even in normal cells. Recently, phytochemicals are considerably advocated as rich sources of anti-cancer agents that deserve more rigorous and valuable investigations. Regarding this issue, several studies suggested phytochemicals such as curcumin [14], resveratrol [32] and capsaicin [33] as potent anti-cancer agents for multiple myeloma treatment. We also found that genipin [34] and icariside II [35] could be applied for multiple myeloma therapy by inducing apoptosis and targeting the signaling molecules such as STAT3.

The STAT proteins were identified for the last decade as latent cytoplasmic transcription factors in response to all cytokine driven signaling [15]. Seven mammalian STAT proteins such as STAT1, 2, 3, 4, 5a, 5b and 6 act as multifunctional mediators to regulate various cellular processes such as cell proliferation, differentiation, angiogenesis, and apoptosis [36]. Especially, STAT3 is constitutively activated in many human cancers, including prostate cancer [37], breast cancer [38], squamous cell carcinoma of the head and neck (SCCHN) [39], nasopharyngeal carcinoma and multiple myelomas [40]. Thus, STAT3 is considered as a valuable therapeutic target molecule for cancer treatment.

Ergosterol peroxide (EP) is a steroid derivative isolated from medicinal mushroom [41]. Several studies reported anti-cancer activity of EP in various types of cancer cells. For instance, Russo and colleagues reported that EP attenuated cell growth and induced apoptosis in human prostate cancer LNCaP and DU-145 cells [42]. Kobori and colleagues reported that EP suppressed inflammatory response in RAW264.7 macrophages and growth of HT29 colon adenocarcinoma cells [43]. Also, Chen and colleagues suggested that EP from the fermentation mycelia of *Ganoderma lucidum *cultivated in the medium exerted the cytotoxic effect against Hep 3B cells [44]. In the present study, we found that EP exerts anti-cancer activity through the inhibition of angiogenesis by targeting the STAT3 signaling pathway in U266 cells *in vitro *and *in vivo*.

EP suppressed constitutive activation of STAT3 in U266, SCC4, DU145 and MDA-MB-231 cells. EP also inhibited the STAT3-DNA binding activity and the nuclear translocation of STAT3, suggesting that EP can prevent STAT3 activation at the transcriptional level. The inhibitory effect of EP on STAT3 activation was partly associated with the inhibition of upstream kinases JAK2 and Src by EP treatment. Furthermore, protein tyrosine phosphatases (PTPs) are known to be implicated in STAT3 signaling, including SHP-1 [45], SHP-2 [46], PTEN [47], SOCS-1 [48] and so on. Our results revealed that EP remarkably enhanced the expression of SHP-1 at levels of protein and mRNA. In contrast, EP had no significant effect on other PTPs such as SHP-2 and PTEN (data not shown). To confirm the significance of SHP-1, we utilized PTP inhibitor pervanadate or SHP-1 siRNA to block the expression of SHP-1. As expected, EP failed to inhibit STAT3 activation in the presence of pervanadate or SHP-1 siRNA, supporting that SHP-1 plays a critical role in dephoshophorylating STAT3 by EP in U266 cells.

Recently, Niu and colleagues reported that constitutive activity of STAT3 up-regulated VEGF expression and tumor angiogenesis [49]. Wei and colleagues also reported that overexpression of constitutively activated mutant STAT3 sufficiently increased VEGF expression and tumor angiogenesis *in vivo *[50]. In contrast, dominant negative STAT3 mutant inhibited VEGF expression as well as angiogenesis. Additionally, it is of interest that STAT3 activation by its upstream Src regulates VEGF mediated angiogenesis and conversely blocking STAT3 inhibits Src-induced VEGF expression [51]. Consistently, in the present study, EP significantly prevented VEGF-induced phosphorylation of STAT3 as well as VEGF-mediated tube formation in HUVECs, indicating anti-angiogenic activity of EP by inhibiting STAT3 phosphorylation. Furthermore, we confirmed anti-tumor effect of EP in mouse xenograft tumor model. Consistent with the results of *in vitro *experiments, EP decreased U266 tumor growth as well as suppressed the expression levels of phospho-STAT3 and CD34 by immunohistochemistry. Although there are evidences that EP induces apoptosis at concentrations of 12.5-50 μM in LNCaP and DU-145 cells for 72 h [41,42,52], in the current study, EP did not show any cytotoxicity against U266 cells for 24 h up to 50 μM. Actually, immunohistochemistry revealed that TUNEL positive cells were increased in EP treated tumor sections, implying that EP can exert anti-angiogenic activity at nontoxic concentrations and possibly induce apoptosis only after long term culture or at high doses. Thus, mechanism and pharmacokinetic studies with EP are still required *in vitro *and *in vivo *to elucidate the relationship between its anti-angiogenic and apoptotic activities at different doses in the near future

## Conclusions

Our findings demonstrate that EP can exert antitumor activity in multiple myeloma U266 cells by anti-angiogenic activity targeting JAK2/STAT3 signaling pathway as a potent anti-cancer agent for multiple myeloma treatment.

## Competing interests

The authors declare that they have no competing interests.

## Authors' contributions

YHR SJJ and SHK SA designed the research studies; YHR, HJeL and HJuL carried out the experiments; YHR SJJ and SHK analyzed and interpreted the data; YHR SJJ and SHK wrote the draft of the manuscript. All authors read and approved the final manuscript.

## Pre-publication history

The pre-publication history for this paper can be accessed here:

http://www.biomedcentral.com/1471-2407/12/28/prepub

## References

[B1] Kim DS Baek NI Oh SR Jung KY Lee IS Kim JH Lee HK Anticomplementary activity of ergosterol peroxide fromNaematoloma fasciculare and reassignment of NMR data Arch Pharm Res 1997 20 3 201 205 10.1007/BF02976145 18975152

[B2] Piovano M Guzman G Garbarino JA Chamy MC The chemistry of Bacidia stipata (Lichens, Ascomycotina) Biochem System Ecol 1999 27 663 664 10.1016/S0305-1978(98)00120-3

[B3] Arisawa M Fujita A Saga M Fukumura H Hayashi T Shimizu M Morita N Three new lanostanoids from Ganoderma lucidum J Nat Prod 1986 49 4 621 625 10.1021/np50046a010 3783158

[B4] Sgarbi DB da Silva AJ Carlos IZ Silva CL Angluster J Alviano CS Isolation of ergosterol peroxide and its reversion to ergosterol in the pathogenic fungus Sporothrix schenckii Mycopathologia 1997 139 1 9 14 10.1023/A:1006803832164 9511231

[B5] Bok JW Lermer L Chilton J Klingeman HG Towers GH Antitumor sterols from the mycelia of Cordyceps sinensis Phytochemistry 1999 51 7 891 898 10.1016/S0031-9422(99)00128-4 10423860

[B6] Lindequist U Teuscher E Wolf B Volsgen A Hoffmann S Kutschabsky L Franke P Seefeldt R [Isolation, characterization and structure elucidation of a substance with immunosuppressive action from Tricholoma populinum Lange] Pharmazie 1989 44 2 165 2748702

[B7] Fujimoto H Nakayama M Nakayama Y Yamazaki M Isolation and characterization of immunosuppressive components of three mushrooms, Pisolithus tinctorius, Microporus flabelliformis and Lenzites betulina Chem Pharm Bull (Tokyo) 1994 42 3 694 697 10.1248/cpb.42.694 8004718

[B8] Kreisel H Lindequist U Horak M Distribution, ecology, and immunosuppressive properties of Tricholoma populinum (Basidiomycetes) Zentralbl Mikrobiol 1990 145 5 393 396 2220166

[B9] Lindequist U Lesnau A Teuscher E Pilgrim H [The antiviral action of ergosterol peroxide] Pharmazie 1989 44 8 579 580 2594833

[B10] Yasukawa K Akihisa T Kanno H Kaminaga T Izumida M Sakoh T Tamura T Takido M Inhibitory effects of sterols isolated from Chlorella vulgaris on 12-0-tetradecanoylphorbol-13-acetate-induced inflammation and tumor promotion in mouse skin Biol Pharm Bull 1996 19 4 573 576 10.1248/bpb.19.573 8860961

[B11] Darnell JE Jr STATs and gene regulation Science 1997 277 5332 1630 1635 10.1126/science.277.5332.1630 9287210

[B12] Ihle JN Cytokine receptor signalling Nature 1995 377 591 594 10.1038/377591a0 7566171

[B13] Zhong Z Wen Z Darnell JE Jr Stat3: a STAT family member activated by tyrosine phosphorylation in response to epidermal growth factor and interleukin-6 Science 1994 264 5155 95 98 10.1126/science.8140422 8140422

[B14] Bharti AC Donato N Aggarwal BB Curcumin (diferuloylmethane) inhibits constitutive and IL-6-inducible STAT3 phosphorylation in human multiple myeloma cells J Immunol 2003 171 7 3863 3871 14500688 10.4049/jimmunol.171.7.3863

[B15] Buettner R Mora LB Jove R Activated STAT signaling in human tumors provides novel molecular targets for therapeutic intervention Clin Cancer Res 2002 8 4 945 954 11948098

[B16] Schreiner SJ Schiavone AP Smithgall TE Activation of STAT3 by the Src family kinase Hck requires a functional SH3 domain J Biol Chem 2002 277 47 45680 45687 10.1074/jbc.M204255200 12244095

[B17] Kim ES Hong SY Lee HK Kim SW An MJ Kim TI Lee KR Kim WH Cheon JH Guggulsterone inhibits angiogenesis by blocking STAT3 and VEGF expression in colon cancer cells Oncol Rep 2008 20 6 1321 1327 19020709

[B18] Lin CM Shyu KG Wang BW Chang H Chen YH Chiu JH Chrysin suppresses IL-6-induced angiogenesis via down-regulation of JAK1/STAT3 and VEGF: an in vitro and in ovo approach J Agric Food Chem 2010 58 11 7082 7087 10.1021/jf100421w 20443595

[B19] Chen Z Han ZC STAT3: a critical transcription activator in angiogenesis Med Res Rev 2008 28 2 185 200 10.1002/med.20101 17457812

[B20] Krzyczkowskia W Malinowska E Suchocki P Kleps J Olejnik M Herold F Isolation and quantitative determination of ergosterol peroxide in various edible mushroom species Food Chemistry 2009 113 1 351 355 10.1016/j.foodchem.2008.06.075

[B21] Jaffe EA Nachman RL Becker CG Minick CR Culture of human endothelial cells derived from umbilical veins. Identification by morphologic and immunologic criteria J Clin Invest 1973 52 11 2745 2756 10.1172/JCI107470 4355998 PMC302542

[B22] Yu CL Meyer DJ Campvell GS Enhanced DNA-binding activity of a stat3-regulated protein in cells transformed by the src oncoprotein Science 1995 269 81 83 10.1126/science.7541555 7541555

[B23] Grant DS Kinsella JL Fridman R Auerbach R Piasecki BA Yamada Y Zain M Kleinman HK Interaction of endothelial cells with a laminin A chain peptide (SIKVAV) in vitro and induction of angiogenic behavior in vivo J Cell Physiol 1992 153 3 614 625 10.1002/jcp.1041530324 1280280

[B24] Ihle JN STATs: signal transducers and activators of transcription Cell 1996 84 331 334 10.1016/S0092-8674(00)81277-5 8608586

[B25] Oka T Ouchida M Koyama M Ogama Y Takada S Nakatani Y Tanaka T Yoshino T Hayashi K Ohara N Gene silencing of the tyrosine phosphatase SHP1 gene by aberrant methylation in leukemias/lymphomas Cancer Res 2002 62 22 6390 6394 12438221

[B26] Han Y Amin HM Franko B Frantz C Shi X Lai R Loss of SHP1 enhances JAK3/STAT3 signaling and decreases proteosome degradation of JAK3 and NPM-ALK in ALK+ anaplastic large-cell lymphoma Blood 2006 108 8 2796 2803 10.1182/blood-2006-04-017434 16825495

[B27] Wu C Sun M Liu L Zhou GW The function of the protein tyrosine phosphatase SHP-1 in cancer Gene 2003 306 1 12 12657462 10.1016/s0378-1119(03)00400-1

[B28] Wang Z Luo F Li L Yang L Hu D Ma X Lu Z Sun L Cao Y STAT3 activation induced by Epstein-Barr virus latent membrane protein1 causes vascular endothelial growth factor expression and cellular invasiveness via JAK3 And ERK signaling Eur J Cancer 2010 46 16 2996 3006 10.1016/j.ejca.2010.07.008 20709526

[B29] Yu H Jove R The STATs of cancer--new molecular targets come of age Nat Rev Cancer 2004 4 2 97 105 10.1038/nrc1275 14964307

[B30] Ebrahem Q Minamoto A Hoppe G Anand-Apte B Sears JE Triamcinolone acetonide inhibits IL-6- and VEGF-induced angiogenesis downstream of the IL-6 and VEGF receptors Invest Ophthalmol Vis Sci 2006 47 11 4935 4941 10.1167/iovs.05-1651 17065510

[B31] Haematology UmfBCfSi: Diagnosis and management of multiple myeloma Br J Haematol 2001 115 3 522 540 11736932 10.1046/j.1365-2141.2001.03206.x

[B32] Bhardwaj A Sethi G Vadhan-Raj S Bueso-Ramos C Takada Y Gaur U Nair AS Shishodia S Aggarwal BB Resveratrol inhibits proliferation, induces apoptosis, and overcomes chemoresistance through down-regulation of STAT3 and nuclear factor-kappaB-regulated antiapoptotic and cell survival gene products in human multiple myeloma cells Blood 2007 109 6 2293 2302 10.1182/blood-2006-02-003988 17164350

[B33] Bhutani M Pathak AK Nair AS Kunnumakkara AB Guha S Sethi G Aggarwal BB Capsaicin is a novel blocker of constitutive and interleukin-6-inducible STAT3 activation Clin Cancer Res 2007 13 10 3024 3032 10.1158/1078-0432.CCR-06-2575 17505005

[B34] Lee JC Ahn KS Jeong SJ Jung JH Kwon TR Rhee YH Kim SH Kim SY Yoon HJ Zhu S Signal transducer and activator of transcription 3 pathway mediates genipin-induced apoptosis in U266 multiple myeloma cells J Cell Biochem 2011 in press 10.1002/jcb.23077 21344490

[B35] Kim SH Ahn KS Jeong SJ Kwon TR Jung JH Yun SM Han I Lee SG Kim DK Kang M Janus activated kinase 2/signal transducer and activator of transcription 3 pathway mediates icariside II-induced apoptosis in U266 multiple myeloma cells Eur J Pharmacol 2011 654 1 10 16 10.1016/j.ejphar.2010.11.032 21172343

[B36] Zhang DW Cheng Y Wang NL Zhang JC Yang MS Yao XS Effects of total flavonoids and flavonol glycosides from Epimedium koreanum Nakai on the proliferation and differentiation of primary osteoblasts Phytomedicine 2008 15 1-2 55 61 10.1016/j.phymed.2007.04.002 17482445

[B37] Mora LB Buettner R Seigne J Diaz J Ahmad N Garcia R Bowman T Falcone R Fairclough R Cantor A Constitutive activation of Stat3 in human prostate tumors and cell lines: direct inhibition of Stat3 signaling induces apoptosis of prostate cancer cells Cancer Res 2002 62 22 6659 6666 12438264

[B38] Dolled-Filhart M Camp RL Kowalski DP Smith BL Rimm DL Tissue microarray analysis of signal transducers and activators of transcription 3 (Stat3) and phospho-Stat3 (Tyr705) in node-negative breast cancer shows nuclear localization is associated with a better prognosis Clin Cancer Res 2003 9 2 594 600 12576423

[B39] Nagpal JK Mishra R Das BR Activation of Stat-3 as one of the early events in tobacco chewing-mediated oral carcinogenesis Cancer 2002 94 9 2393 2400 10.1002/cncr.10499 12015764

[B40] Hsiao JR Jin YT Tsai ST Shiau AL Wu CL Su WC Constitutive activation of STAT3 and STAT5 is present in the majority of nasopharyngeal carcinoma and correlates with better prognosis Br J Cancer 2003 89 2 344 349 10.1038/sj.bjc.6601003 12865928 PMC2394270

[B41] Takei T Yoshida M Ohnishi-Kameyama M Kobori M Ergosterol peroxide, an apoptosis-inducing component isolated from Sarcodon aspratus (Berk.) S. Ito Biosci Biotechnol Biochem 2005 69 1 212 215 10.1271/bbb.69.212 15665489

[B42] Russo A Cardile V Piovano M Caggia S Espinoza CL Garbarino JA Pro-apoptotic activity of ergosterol peroxide and (22E)-ergosta-7, 22-dien-5alpha-hydroxy-3, 6-dione in human prostate cancer cells Chem Biol Interact 2010 184 3 352 358 10.1016/j.cbi.2010.01.032 20100469

[B43] Kobori M Yoshida M Ohnishi-Kameyama M Shinmoto H Ergosterol peroxide from an edible mushroom suppresses inflammatory responses in RAW264.7 macrophages and growth of HT29 colon adenocarcinoma cells Br J Pharmacol 2007 150 2 209 219 10.1038/sj.bjp.0706972 17160010 PMC2042906

[B44] Chen YK Kuo YH Chiang BH Lo JM Sheen LY Cytotoxic activities of 9, 11-dehydroergosterol peroxide and ergosterol peroxide from the fermentation mycelia of ganoderma lucidum cultivated in the medium containing leguminous plants on Hep 3B cells J Agric Food Chem 2009 57 13 5713 5719 10.1021/jf900581h 19492810

[B45] Tenev T Bohmer SA Kaufmann R Frese S Bittorf T Beckers T Bohmer FD Perinuclear localization of the protein-tyrosine phosphatase SHP-1 and inhibition of epidermal growth factor-stimulated STAT1/3 activation in A431 cells Eur J Cell Biol 2000 79 4 261 271 10.1078/S0171-9335(04)70029-1 10826494

[B46] Kim H Baumann H Dual signaling role of the protein tyrosine phosphatase SHP-2 in regulating expression of acute-phase plasma proteins by interleukin-6 cytokine receptors in hepatic cells Mol Cell Biol 1999 19 8 5326 5338 10409724 10.1128/mcb.19.8.5326 PMC84376

[B47] Sun S Steinberg BM PTEN is a negative regulator of STAT3 activation in human papillomavirus-infected cells J Gen Virol 2002 83 Pt 7 1651 1658 12075083 10.1099/0022-1317-83-7-1651

[B48] Norkina O Dolganiuc A Catalano D Kodys K Mandrekar P Syed A Efros M Szabo G Acute alcohol intake induces SOCS1 and SOCS3 and inhibits cytokine-induced STAT1 and STAT3 signaling in human monocytes Alcohol Clin Exp Res 2008 32 9 1565 1573 10.1111/j.1530-0277.2008.00726.x 18616672 PMC4116614

[B49] Niu G Wright KL Huang M Song L Haura E Turkson J Zhang S Wang T Sinibaldi D Coppola D Constitutive Stat3 activity up-regulates VEGF expression and tumor angiogenesis Oncogene 2002 21 13 2000 2008 10.1038/sj.onc.1205260 11960372

[B50] Wei D Le X Zheng L Wang L Frey JA Gao AC Peng Z Huang S Xiong HQ Abbruzzese JL Stat3 activation regulates the expression of vascular endothelial growth factor and human pancreatic cancer angiogenesis and metastasis Oncogene 2003 22 3 319 329 10.1038/sj.onc.1206122 12545153

[B51] Inoue S Branch CD Gallick GE Chada S Ramesh R Inhibition of Src kinase activity by Ad-mda7 suppresses vascular endothelial growth factor expression in prostate carcinoma cells Mol Ther 2005 12 4 707 715 10.1016/j.ymthe.2005.05.015 16054437

[B52] Kobori M Yoshida M Ohnishi-Kameyama M Takei T Shinmoto H 5alpha,8alpha-Epidioxy-22E-ergosta-6, 9(11),22-trien-3beta-ol from an edible mushroom suppresses growth of HL60 leukemia and HT29 colon adenocarcinoma cells Biol Pharm Bull 2006 29 4 755 759 10.1248/bpb.29.755 16595913

